# Comparison of shear bond strength of different types of intracanal posts in restoring extensively damaged primary anterior teeth

**DOI:** 10.34172/joddd.39232

**Published:** 2024-06-24

**Authors:** Shabnam Asghari Mollabashi, Shima Nourmohamadi, Afrooz Nakhostin

**Affiliations:** ^1^Students Research Committee, Arak University of Medical Sciences, Arak, Iran; ^2^Department of Pediatric Dentistry, School of Dentistry, Arak University of Medical Sciences, Arak, Iran; ^3^Department of Restorative Dentistry, School of Dentistry, Arak University of Medical Sciences, Arak, Iran

**Keywords:** Composite resin post, Glass fiber post, reverse metal post, shear bond strength

## Abstract

**Background.:**

Severe caries in early childhood is a concern for both children receiving dental treatment and their parents. This dental disease progresses rapidly and quickly damages the coronal part of the tooth. When there is insufficient coronal structure to support a coronal restoration, using intracanal components following root canal treatment increases tooth resistance and helps provide retention for the coronal restoration. This study compared the shear bond strength of three types of intracanal posts (composite resin post, reverse metal post, and fiber post) in severely damaged primary anterior teeth.

**Methods.:**

This in vitro study was conducted on 30 extracted anterior primary teeth with at least two-thirds of healthy roots and no prior pulp treatment. The teeth were randomly divided into three groups of 10: group 1: composite resin post with 8th generation universal bonding, group 2: reverse metal post with GC restorative glass cement, and group 3: fiber post with GC restorative glass cement. After placing the post, the samples were restored with a height of 3 mm from cementoenamel junction (CEJ) using an Anterior GC Gradia Packable composite resin. All the samples underwent 500 cycles of thermocycling in a hot water bath at 55±2 °C and a cold water bath at 5±2 °C. The shear strength of the samples was then evaluated using an electromechanical universal testing machine at a rate of 1 mm/min and at a location 2 mm coronal to the CEJ in terms of megapascals.

**Results.:**

The average shear bond strength of composite resin posts with 8th generation bonding application was 8.02220 MPa, reverse metal posts with glass ionomer application was 13.8600 MPa, and fiber posts with glass ionomer application was 9.7400 MPa.

**Conclusion.:**

Based on these findings, it can be concluded that the highest shear bond strength in this study was related to the reverse metal post, and the lowest shear bond strength was related to the composite resin post. According to the results, reverse metal posts demonstrated better shear bond strength than composite resin posts and fiber posts (*P*<0.05).

## Introduction

 Severe caries in early childhood are a concern for both children undergoing dental treatment and their parents.^1^ This dental disease progresses rapidly, quickly destroying the coronal structure of the tooth.^2^ Reconstructing severely damaged primary anterior teeth, particularly in young children, remains one of the most difficult challenges in pediatric dentistry. The patients’ small size and young age make the restorative process difficult.^3^ Early loss of these teeth can lead to chewing and phonetic problems, delays in premaxilla development, speech impairment, malocclusion, prevalence of parafunctional habits, and psychological issues affecting the child’s self-confidence. Therefore, tooth restoration and reconstruction should be carried out despite the challenges.^4^

 When there is insufficient coronal structure to support the restoration, using retainer components within the root canal after canal treatment increases tooth resistance and helps provide a retainer for coronal restoration.^5^ However, the physiological resorption of primary tooth roots can interfere with the placement of retention-providing components within the root canal. Consequently, the entire length of the primary tooth root canal cannot be used to provide retention. Therefore, all the methods of providing a clamp within the primary tooth root canal are generally limited to the coronal third of the root canal.^6^ Posts used in primary teeth can be made of metal, biological materials, omega or alpha orthodontic wires, composite resins, or fiber posts.^1^

 Since 1998, composite resin posts have been used in restoring severely degraded primary teeth. These posts can be used confidently if there is proper chewing function, a balanced diet, and oral hygiene control.^7^ In recent years, much attention has been directed toward using fiber posts to increase retention in primary teeth. One of the obvious advantages of prefabricated fiber posts is the chemical and physical bonding to the restorative material, which reduces the risk of primary root fracture and does not alter the restoration color.^8^

 Recently, using an inverted metal pin as a retainer within the root canal in anterior primary teeth has been highly considered. The space required for this type of metal post is 3 mm coronally, cut in a square shape, with the screw part positioned towards the crown like a post, creating a strong and durable grip for the final restoration.^9^ In addition to the type of post, the type of bonding used is crucial. An effective bond reduces marginal microleakage, bacterial penetration of secondary caries, postoperative sensitivity, and the possibility of pulp inflammation, and it preserves tooth structure with minimal cavity preparation. Eighth-generation universal bonding can create a suitable bond with wet and dry surface dentin. According to the manufacturer of this bonding agent, it has high bond strength. One of their advantages, especially when working with children, is the ease and speed of application and the lack of acid etch use.^10^

 In previous studies, numerous clinical reports have focused on the reconstruction of primary teeth using various types of posts, with one- to two-year follow-up periods to assess the durability of these restorations. However, these restorations’ physical and mechanical properties have primarily been investigated in laboratory settings. As a result, it is essential to conduct further laboratory studies to compare the strength of different methods used for crown reconstruction in anterior primary teeth. This study compared the shear bond strength of three types of intra-canal posts (composite resin, reverse metal, and fiber posts) in severely damaged anterior deciduous teeth.

## Methods

###  Inclusion and exclusion criteria for the study

 In this in vitro study, extracted human primary anterior teeth with caries were selected. All these teeth had at least 2 mm of healthy coronal tissue above the cementoenamel junction (CEJ) and two-thirds of healthy roots without any history of previous pulp treatment. Teeth that did not meet these criteria were excluded. Mandibular central and lateral primary teeth were not chosen due to the thinness of their roots and crowns.

###  Sample selection, treatment, and sampling

 In this study, 30 carious anterior deciduous teeth with at least two-thirds healthy roots and no history of pulp treatment were collected and cleaned of contamination. To disinfect the samples before the laboratory stages of the research, they were immersed in a 0.01% thymol solution for 24 hours. The teeth were then stored in isotonic saline at room temperature. The teeth were standardized according to the established protocols using a cylindrical diamond bur (Tizkavan, Iran). The teeth were cut 1 mm above the CEJ with high speed and air cooling. A maximum of five teeth were cut per bur. The access cavity was prepared, and the root canal was 1 mm shorter than the root length. An attempt was made to allow the same cavity wall and tooth structure thickness in each group using an orthometer gauge (Korkhaus Orthometer Kit, 75228 Ispringen, Dentaurm, Germany). The root canal was cleared using the step-back technique with K-files from #20 to #40. After each file, a 0.9% isotonic serum solution was used for irrigation, and after cleaning with a paper point, the root canal was dried using a #40 paper point.

**Figure 1:**
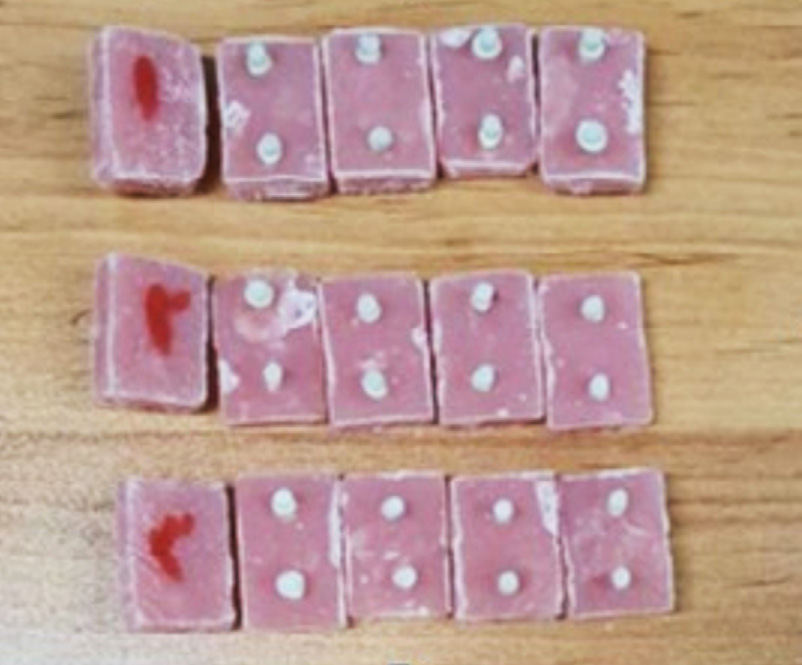
Samples mounted in Acropars 200 cold-cured acrylic resin, Iran

 After root canal preparation, it was treated with Zoliran zinc oxide‒eugenol (ZOE, Iran), filled with paste consistency, and the 4-mm apical level of the root canal was evacuated. Then, a 1-mm-thick polycarboxylate base was placed over it to prevent the negative effect of ZOE on the composite resin setting. After the base had set, excess polycarboxylate was removed from the root canals and the pulp chamber space using a round carbide bur. All the samples were then mounted within self-cured acrylic resin (Acropars 200 Cold-cured Acrylic Resin, Iran) with a diameter of 2 cm and a height of 4 cm (according to the clamp of the shear bond strength measuring device). The teeth were positioned so their entire crowns remained outside the acrylic resin (Figure 1).

 The teeth were randomly placed and divided into three groups of 10, numbered from 1 to 30, and restored using three types of posts within the root canal.

- Group 1: Teeth restored with a composite resin post (Gradia Direct Universal X composite; GC, Tokyo, Japan) and the application of an 8th-generation universal bonding agent (G-Premio Bond; GC, Tokyo, Japan)
- Group 1: Teeth restored with an inverted metal post using GC glass restorative cement (GC, Tokyo, Japan)
- Group 3: Teeth restored with a fiber post using GC glass cement (GC, Tokyo, Japan)

###  Group 1

 In group 1, the teeth were cleaned, and excess moisture was removed using gentle air pressure (without water and oil). The surface was then prepared. In the next step, an 8th-generation bonding agent (G-Premio Bond; GC, Tokyo, Japan) was applied to the dentin surface according to the manufacturer’s instructions using a cotton applicator (Cavibrush-FGM, Brazil). After evaporating the solvent with 5 seconds of mild air pressure, the bonding agent was cured using a Guilin light-curing device (Woodpecker ME, Dycal Instrument Co., China) with an output intensity of 1200 mW/cm^2^ for 10 seconds.

**Figure 2:**
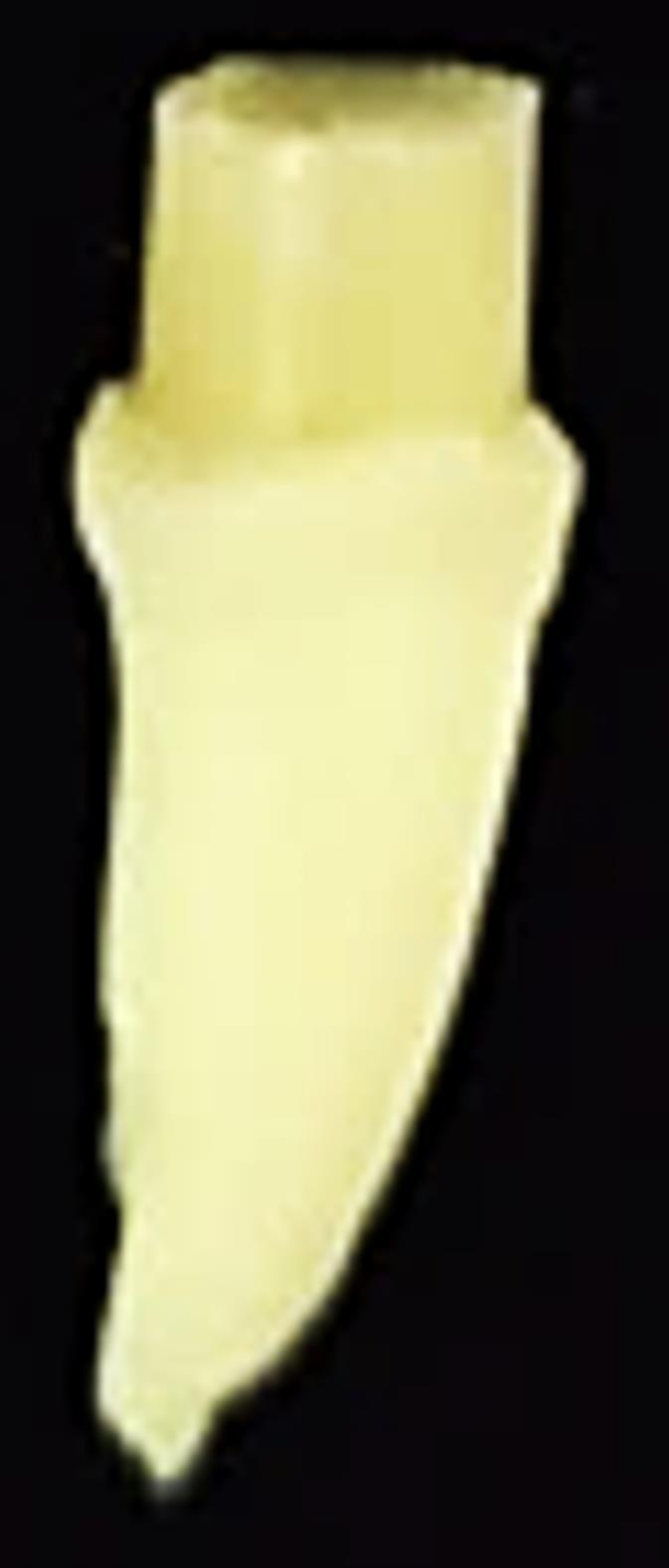
Anterior primary tooth restored with composite resin post

 Next, a composite resin (Gradia Direct Universal X; GC, Tokyo, Japan) was shaped into 1.5‒2-mm pieces to create a composite resin post within the root canal space, placed on a base polycarboxylate, and condensed to achieve contact. After ensuring the composite resin was in contact with the dentin surface, each layer was cured separately for 40 seconds using the light-curing device, ensuring proper contact with the floor and walls. To equalize the cross-section of the samples, a transparent plastic tube with a diameter of 4 mm and a height of 3 mm was used, along with the same composite resin for crown reconstruction in layers. After re-curing from the buccal and lingual sides, the plastic tube was cut and removed with a scalpel (Figure 2).

###  Group 2 

 In this group, the root canal was nearly square, with a semi-rounded line angle to match the square head of the metal post. The metal post head’s line angle was beveled to reduce stress on the dentinal walls of the buccal root canal, and the proper placement of the metal post in the canal was ensured. The length of the screw portion of the canal, before placement, was shortened by 2 mm. The metal post was cleaned, thoroughly dried, and repositioned. It was placed inside the root canal so that the entire 3 mm length of the post head part was within the root canal, and the screw portion of the post was positioned 3 mm outside the root canal.

**Figure 3:**
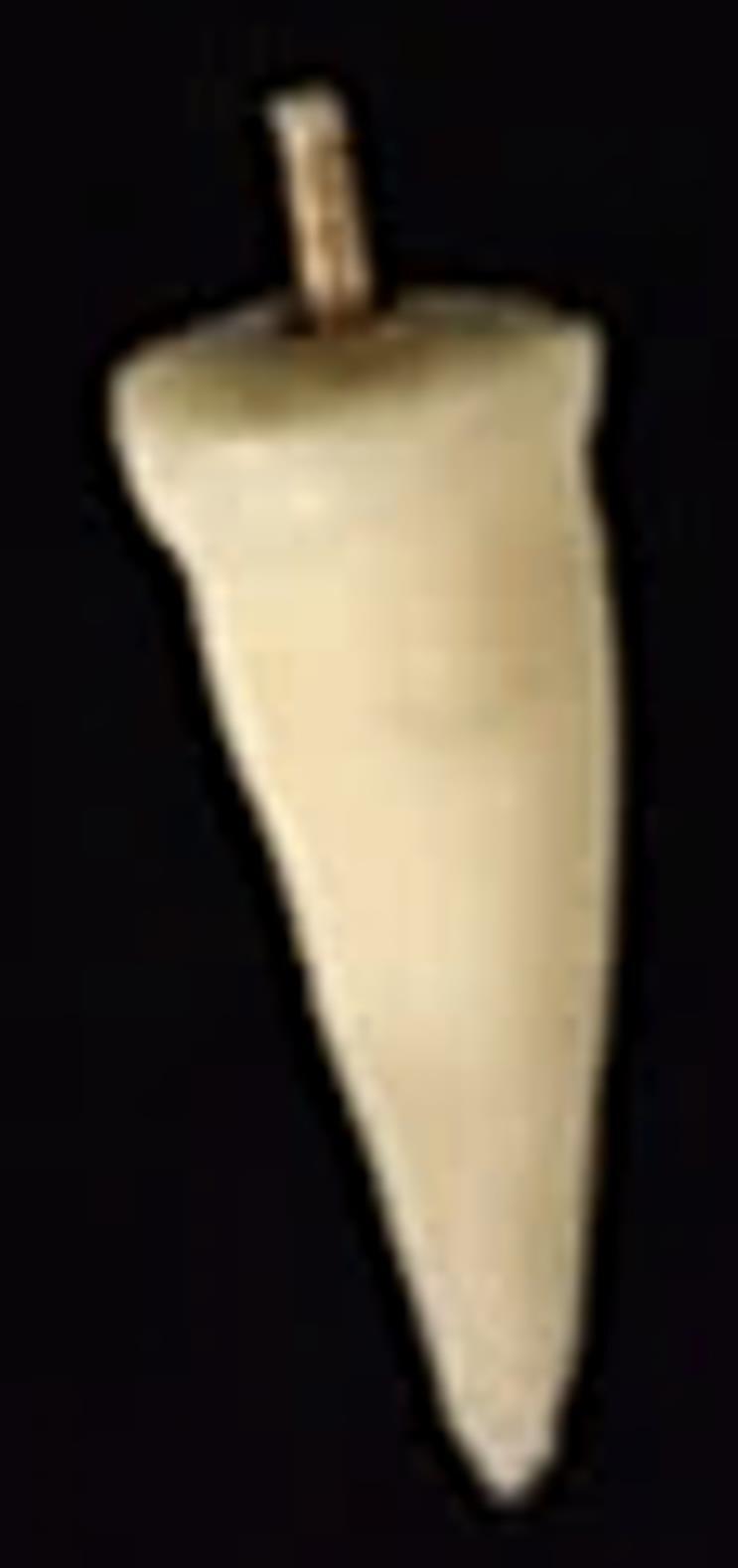
Inverted metal post

 The post was cemented using GC glass ionomer restorative cement (GC, Tokyo, Japan), and the tooth structure and grooved part of the metal post were bonded using the 8th-generation universal bonding agent (G Premio Bond). They were then cured for 10 seconds using a light-curing device (GC, Tokyo, Japan; Guilin Woodpecker Medical Instrument Co., China). The tooth crown was reconstructed with composite resin (Gradia Direct Universal X; GC, Tokyo, Japan). Similar to group 1, the matching and shaping were performed in layers and with the help of a plastic tube around the screw part of the metal post (Figure 3).

###  Group 3 

**Figure 4:**
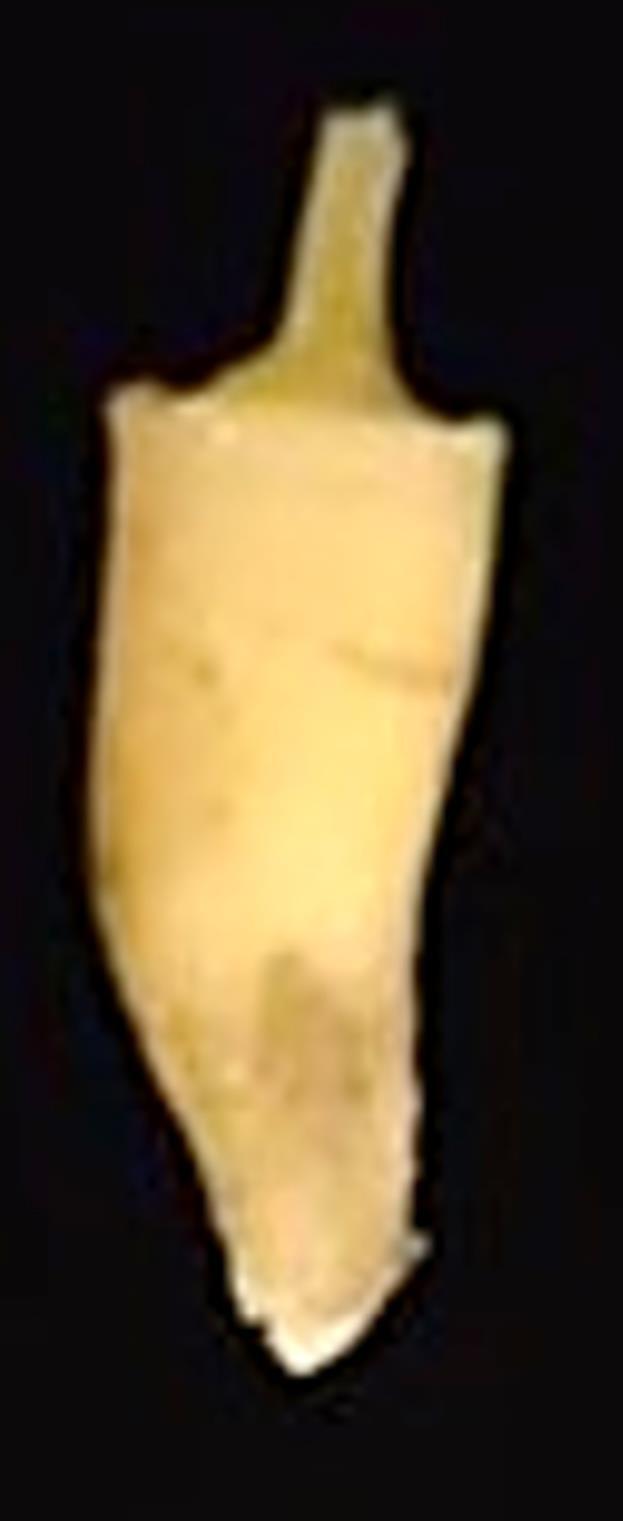
Glass fiber post

 In this group, a #1 glass fiber post with a length of 6 mm was cut (3 mm to be placed inside the root canal and the remaining 3 mm to reinforce the reconstructed crown). The length of the fiber post was measured and checked using a numbered probe before cementing it inside the root canal. The glass fiber post was cemented using GC glass restorative cement (GC, Tokyo, Japan) with a powder-to-liquid ratio of 1:2 (one scoop of powder and two drops of liquid), per the manufacturer’s instructions. The crown was reconstructed using composite resin (Composite Gradia Direct Universal X; GC, Tokyo, Japan), similar to groups 1 and 2 (Figure 4).

 The samples were stored in distilled water at room temperature to prevent dehydration. To simulate thermal changes in the mouth, all the samples underwent 500 cycles of thermocycling in a hot water bath at 55 ± 2 ºC and a cold water bath at 5 ± 2 ºC. The dwell time in each temperature was 30 seconds. The shear bond strength of the samples was determined using a Zwick Roell Z050 universal testing machine at a crosshead speed of 1 mm/min. The evaluation was conducted at a point 2 mm incisal to the CEJ and measured in megapascals.

###  Data analysis

 To compare the bond strength between the three groups, the Kruskal-Wallis test was used for non-normal data, while analysis of variance (ANOVA) was applied to normal data. The normality of the data was evaluated using the Kolmogorov-Smirnov test. Additionally, the frequency of variables across the three groups was compared using the chi-squared test. All statistical tests were performed at the 95% confidence level, employing Stata version 11 statistical software.

## Results

 The present study investigated the shear bond strength of three post types inside the channel – composite resin post, reverse metal post, and glass fiber post – in restoring severely damaged anterior primary teeth. According to the Kolmogorov-Smirnov test, which was used to determine the normality of all the groups, all the samples had a normal distribution, and a parametric test was used for the samples.

**Figure 5:**
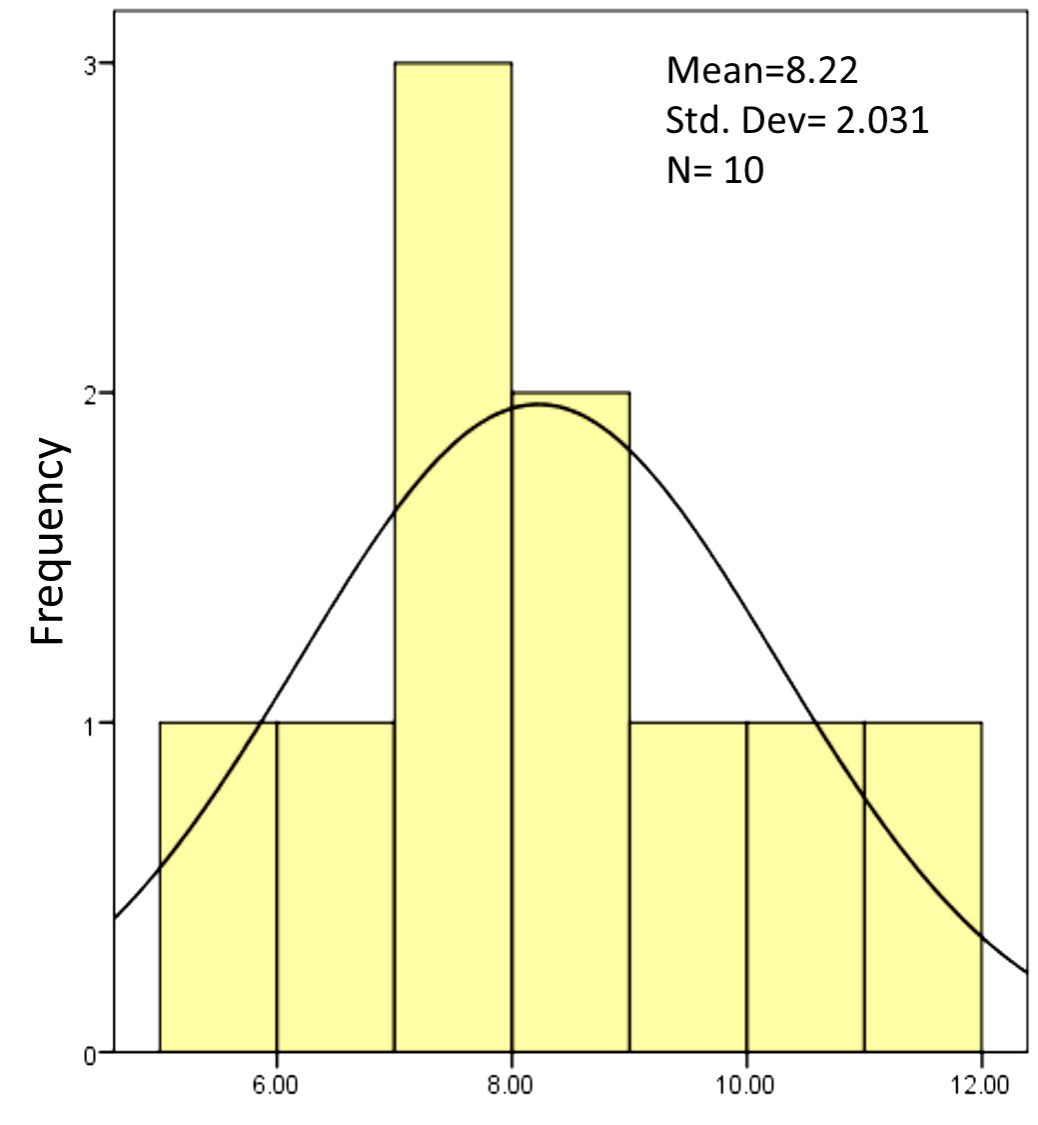
Shear bond strength level of the first group (composite post)

**Figure 6:**
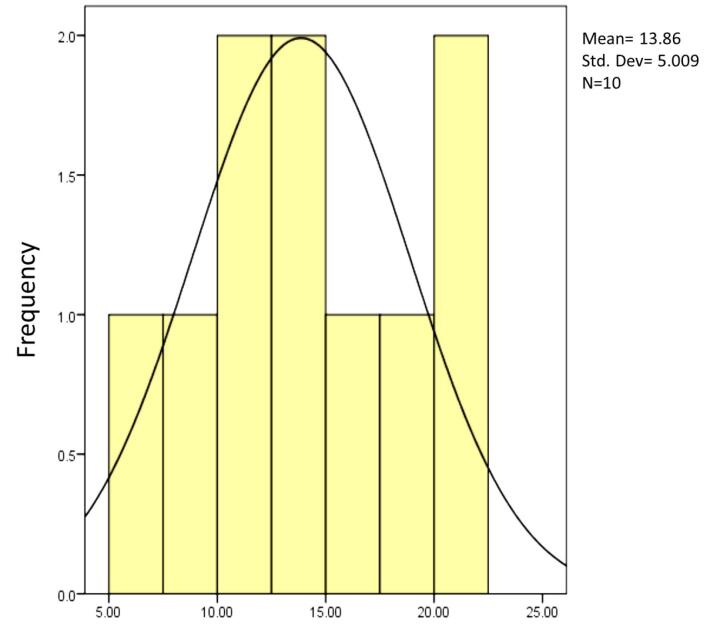
The level of shear bond strength of the second group (inverted brass post)

**Figure 7:**
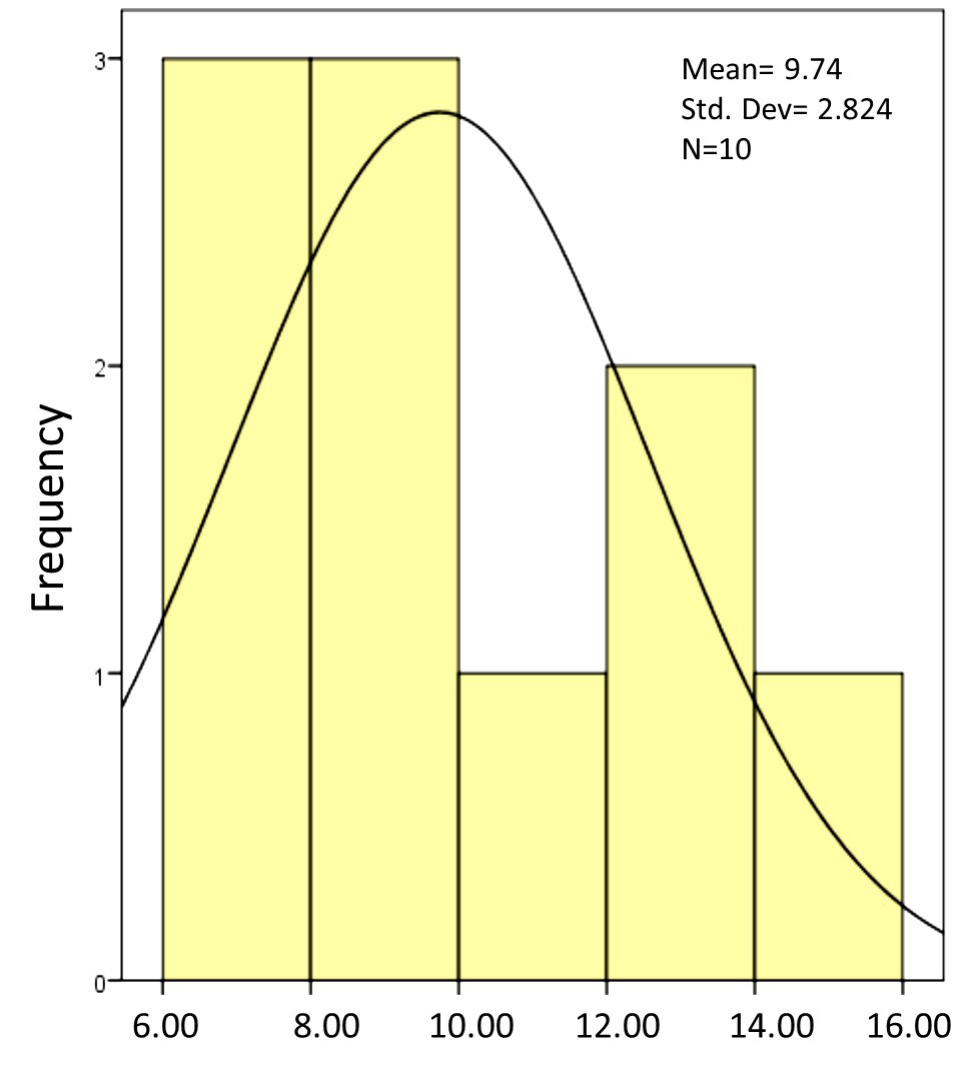
The shear bond strength of the third group (fiber post)

 The results showed significant differences between groups 1 and 2 and groups 2 and 3, wit

 h no significant difference between groups 1 and 3 (Table 1 and Figures 5 to 7).

**Table 1:** Shear strength level in all three groups (MPa)

**Number of samples**	**First group: Post** **Composite**	**Second group: brass post reverse**	**The third group: fiber post**
1	11.92	11.04	12.86
2	7.17	21.33	6.81
3	8.81	13.86	9.57
4	10.26	18.03	11
5	7.39	8.83	8.28
6	5.04	11.54	14.23
7	6.04	5.23	9.45
8	9.53	13.19	12.55
9	7.54	20.11	6.44
10	8.52	15.14	6.3
Mean MPa	8.222	13.860	9.74

 The mean shear bond strength of the composite resin post using the 8th-generation bonding agent was 8.2220 MPa, that of the reverse metal post using glass ionomer was 13.8600, and that of the fiber post using glass ionomer was 9.7400 (Table 2).

**Table 2:** Comparison of mean and standard deviation of shear bond strength in three groups

**First Group**		**Second group**		**Third group**		***P*** ** value**
**Mean**	**SD**	**Mean**	**SD**	**Mean**	**SD**	
8.2220	2.03099	13.860	5.0094	9.740	2.08244	0.05

SD, standard deviation.

 Based on the results, the shear bond strength of the composite resin post, compared to the reverse metal post and the reverse metal post, was significantly different from that of the fiber post, with no significant difference in the shear bond strength between the composite resin post and the fiber post (Table 3).

**Table 3:** Pairwise comparison of mean and standard deviation of shear bond strength in groups

**Number**	**Groups**	**SD**	**Mean**	***P*** ** value**
1	1 and 2	1.57473	5.6380	0.004
2	1 and 3	1.57473	1.5180	0.605
3	2 and 3	1.57473	4.120	0.037

SD, standard deviation.

**Figure 8:**
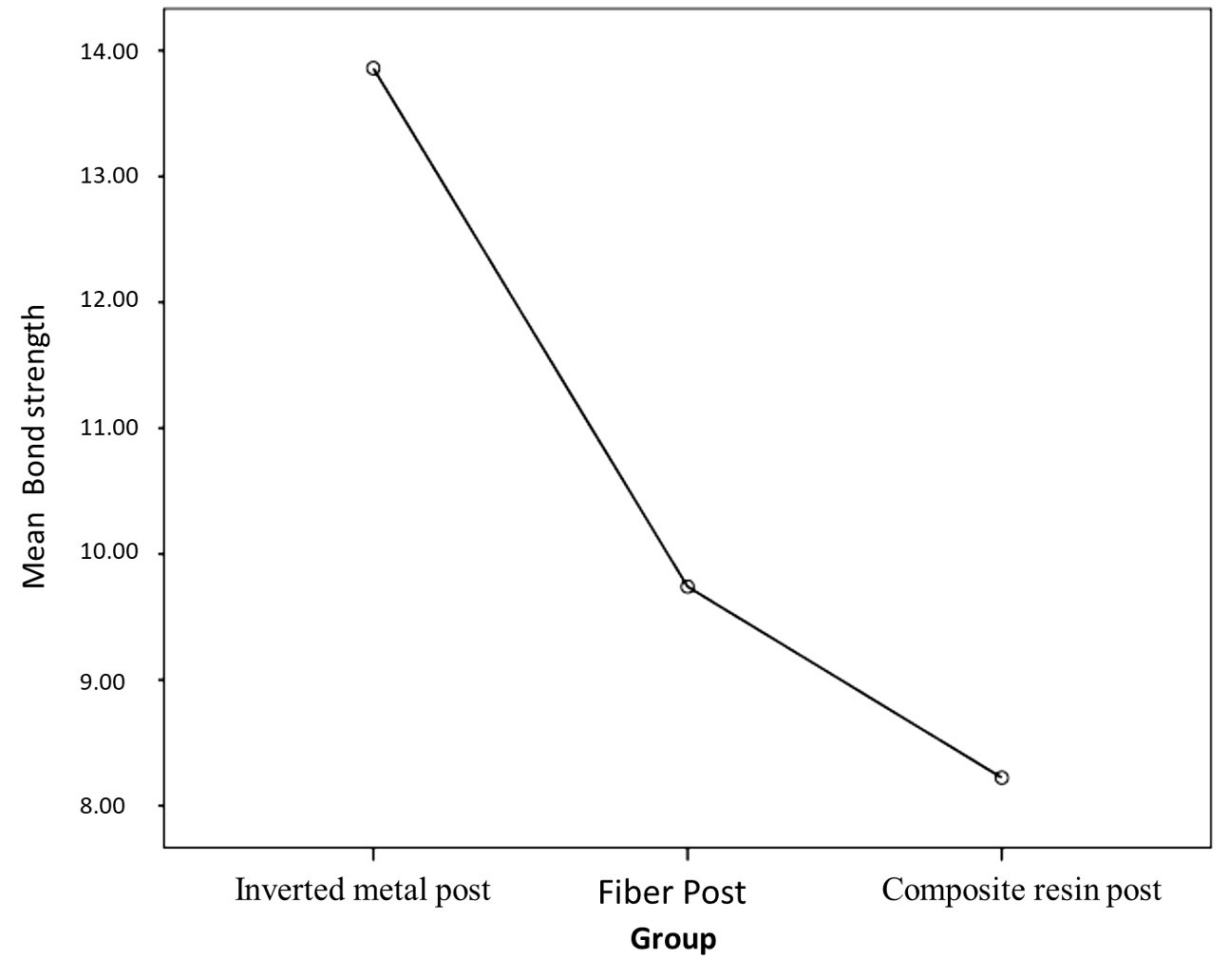
Comparison of the mean shear bond strength of the of three groups

 Based on these results, it can be concluded that the highest shear bond strength in this study was related to the reverse metal post, and the lowest bond strength was related to the composite resin post (Figure 8).

## Discussion

 Based on the results of the present study, it can be concluded that the highest shear bond strength in this study was related to the reverse metal post, and the lowest bond strength was related to the composite resin post.

 This study investigated the shear bond strength of three types of posts (reverse metal post, composite resin post, and fiber post) in treating severely damaged anterior primary teeth. After analyzing the data, the results were as follows: The mean shear bond strength of the composite resin post using the 8th-generation bonding was 8.02220 MPa, that of the reverse metal post using glass ionomer cement was 13.8600 MPa, and that of the fiber post using glass ionomer cement was 9.7400 MPa. Based on these findings, it can be concluded that the highest shear bond strength in this study was related to the reverse metal post, and the lowest shear bond strength was related to the composite post. The null hypothesis of this study stated that there is no significant difference between the shear bond strength of the reverse metal, composite resin, and fiber posts. The results showed significant differences in the shear bond strength between the reverse metal and the composite resin posts and between the reverse metal and fiber posts (*P* < 0.05). However, no significant difference was observed between the composite resin and fiber posts.

 Based on the results of the present study, the reverse metal post demonstrated better shear bond strength than composite resin and fiber posts (*P* < 0.05). This significant difference might be attributed to the physical properties of the metal post itself or the success of the mechanical connection of the reverse metal post to the 3 mm coronal part of the root canal, which was cut in a square shape in primary teeth. This contrasts with the weaker bonding of glass fiber and composite resin posts to the root canal walls of primary teeth, as past studies have also highlighted this difference in these three methods.^11^

 In the study by Kadkhodaei et al,^12^ anterior teeth restored with a glass fiber post proved more resistant to breaking than composite resin posts, suggesting that glass fiber posts can be a promising method for restoring anterior primary teeth. This finding is different from the results of the present study. The difference in the measured property (resistance to fracture) and variations in the cement and composite types used might have contributed to the discrepancy in the findings.

 Additionally, the results of the present study align with Ahmadi’s^1^ research, which indicated that the reverse metal post, compared to the glass fiber post, yields better outcomes in the reconstruction of anterior primary teeth in terms of the survival rate of the final restoration. Oner and Patir Munevveroglu^5^ concluded in a study that glass fiber posts have maximum retention and marginal integrity, followed by polyethylene fiber posts. In contrast, composite resin posts exhibited the lowest retention and marginal integrity. The present study found no significant difference in shear bond strength between composite resin and glass fiber posts, which could be attributed to differences in the study type, sample size, and the cement and composite resin types used.

 The results of this study are different from Vafaei and colleagues’ research,^3^ which demonstrated that the survival of restoration with two types of posts, i.e., glass fiber post and reverse metal post, showed no statistically significant difference. The discrepancy may stem from the different intraoral study conditions, such as saliva and the acidic environment of the oral cavity, as well as variations in the characteristics measured in the two studies.

 Furthermore, the present results align with a study by Eshghi et al,^9^ comparing the tensile bond strength of three types of posts – orthodontic wire, composite resin post, and reversed metal post – in the reconstruction of primary canine crowns. In the above study, the tensile bond strength of the reverse metal post exhibited a significant difference compared to the composite resin post and orthodontic wire, whereas the tensile bond strength of the composite resin post and orthodontic wire showed no significant difference.

 Memarpour et al^6^ reported that the highest value of tensile bond strength was related to composite resin posts with an undercut, followed by polyethylene fiber posts, glass fiber posts with flowable composite resin cement, and glass fiber posts with resin cement. The composite post with an undercut exhibited the highest resistance against dislodgement, but fiber posts cemented with flowable composite resin demonstrated favorable results regarding tensile strength and fracture mode. These findings are different from the results of the present study, which could be attributed to differences in cement type, restoration method, composite resin post composition, and sample size. Pithan et al^13^ concluded in a laboratory study that there were significant differences between composite resin, orthodontic wire, and glass fiber posts. The present study also suggested that the glass fiber post was not superior to the composite resin post. The use of metal posts, on the other hand, raised concerns regarding aesthetics due to their metallic appearance and reduced ability to conform to the tooth’s natural shape, thus allowing for more composite resin material to be placed around the post. However, more aesthetically pleasing results could be achieved by applying a layer of opaque composite resin.^2^

## Conclusion

 The results of the present study showed that using prefabricated metal posts in a reverse shape for the reconstruction of primary anterior teeth provides better shear bond strength than glass fiber and composite resin posts. Although there was no significant difference between composite resin and glass fiber posts, the former offered satisfactory aesthetics. Given its low cost and availability, this type of restoration can be considered a suitable option for reconstructing severely damaged anterior primary teeth. It should be noted that laboratory study conditions, especially in pediatric dentistry, are quite different from actual restorations in a child’s mouth. Factors such as the child’s cooperation level can have a significant impact.

 In clinical studies involving pediatric dentistry, numerous factors can influence outcomes, including a child’s cooperation, age, root dentin condition, psychological conditions, and health habits. These factors make conducting such studies particularly challenging. Therefore, after completing laboratory studies, it is necessary to conduct appropriate clinical studies to evaluate various methods, including the new method presented in this study.

## Acknowledgments

 This article was extracted from a research project approved by the Vice Chancellor for Research of Arak University of Medical Sciences and Health Services. The authors express their gratitude and appreciation to the Vice Chancellor for Research of the Arak University of Medical Sciences.

## Competing Interests

 The authors declare no conflicts of interest in this work.

## Consent for Publication

 Not applicable.

## Data Availability Statement

 The datasets used and/or analyzed during the current study are available from the corresponding author upon reasonable request.

## Ethical Approval

 The current study was conducted according to the Declaration of Helsinki and approved by the Medical Ethics Committee of Arak University of Medical Sciences (IR.ARAKMU.REC.13400.110). The patients signed informed consent forms before participating in the study. The authors certify that the study was performed in accordance with relevant guidelines and regulations.

## Funding

 There was no specific funding for this study.
